# Effect of *CYP2C19* genotypes on tamoxifen metabolism and early-breast cancer relapse

**DOI:** 10.1038/s41598-020-79972-x

**Published:** 2021-01-11

**Authors:** A. B. Sanchez-Spitman, J. J. Swen, V. O. Dezentjé, D. J. A. R. Moes, H. Gelderblom, H. J. Guchelaar

**Affiliations:** 1 Department of Clinical Pharmacy and Toxicology, Leiden University Medical Center, Albinusdreef 2, 2300 RC Leiden, The Netherlands; 2 Leiden Network for Personalised Therapeutics, Leiden University Medical Center, Leiden, The Netherlands; 3 Department of Medical Oncology, Netherlands Cancer Institute/Antoni Van Leeuwenhoek, Amsterdam, The Netherlands; 4 Department of Medical Oncology, Leiden University Medical Center, Leiden, The Netherlands

**Keywords:** Breast cancer, Cancer genetics

## Abstract

CYP2C19*2 and CYP2C19*17 might influence tamoxifen metabolism and clinical outcome. Our aim was to investigate the effect of *CYP2C19* genotypes on tamoxifen concentrations and metabolic ratios (MRs) and breast cancer recurrence in a large cohort of Caucasian women**.** Genetic variants (*CYP2D6* and *CYP2C19* genotypes), tamoxifen and metabolites concentrations, baseline characteristics, and breast cancer recurrence from the CYPTAM study (NTR1509) were used. CYP2C19*2 and CYP2C19*17 genotypes were evaluated as alleles and as groups based on *CYP2D6* genotypes (high, intermediate and low activity). Log-rank test and Kaplan–Meier analysis were used to evaluate differences in recurrence defined as relapse-free survival (RFS). Classification tree analyses (CTAs) were conducted to assess the levels of interactions per polymorphism (*CYP2D6* and *CYP2C19* genotypes) on endoxifen concentrations. No differences in mean concentrations and MRs were observed when comparing *CYP2C19* genotypes (CYP2C19*1/*1; CYP2C19*1/*2; CYP2C19*2/*2; CYP2C19*1/*17; CYP2C19*17/*17; CYP2C19*2/*17). Only significant differences (*p* value < 0.05) in mean concentrations and MRs were observed when comparing tamoxifen activity groups (high, intermediate and low activity). A log-rank test did not find an association across *CYP2C19* genotypes (*p* value 0.898). CTAs showed a significant relationship between *CYP2D6* and endoxifen (*p* value < 0.0001), but no association with *CYP2C19* genotypes was found. *CYP2C19* polymorphisms do not have a significant impact on tamoxifen metabolism or breast cancer relapse.

## Introduction

Worldwide, breast cancer is still the most frequent malignity in women^1,2^, and accounted for 571,000 deaths in 2015^1^. Since the majority of newly diagnosed breast cancer cases are estrogen-receptor positive^1,2^, endocrine therapy with tamoxifen or aromatase inhibitors is recommended^3,4^. For many years, tamoxifen has been prescribed as monotherapy or with subsequent switch to an aromatase inhibitor after 2 or 3 years of endocrine therapy^3,4^. In the adjuvant scenario, tamoxifen therapy decreases mortality and disease recurrences of breast cancer^5^, whilst in the metastatic setting prolonged survival outcomes has been observed^6^. Unfortunately, there is a high variability in tamoxifen response^7^, and about 30% of patients using adjuvant tamoxifen still will have a disease relapse^5^.

**Figure 1:**
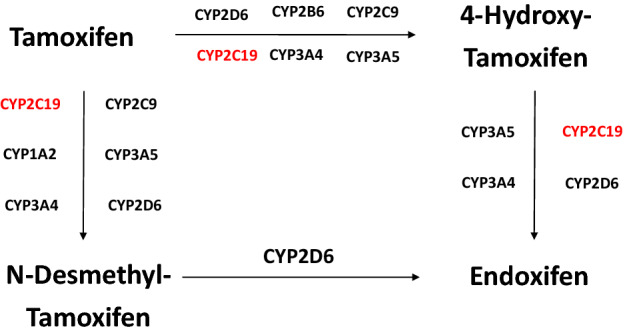
Tamoxifen metabolism.

Tamoxifen is a competitive estrogen receptor antagonist^8,9^ and is metabolized into its primary metabolites, N-desmethyl-tamoxifen (NDM-tamoxifen; Supplementary Table 1) and 4-hydroxy-tamoxifen, followed by a second conversion into endoxifen (Fig. 1)^8–10^. Both 4-hydroxy-tamoxifen and endoxifen have similar anti-estrogenic potencies^11^, but endoxifen is reported as the active metabolite, as it is found in 5–10 times higher concentrations than 4-hydroxy-tamoxifen^8^.

In tamoxifen metabolism, the limiting step in the transformation to endoxifen is regulated by CYP2D6 enzyme^8,9^. Although many studies have associated genetic variants in *CYP2D6* gene with clinical outcome^12^, many other researchers have reported null-association between survival outcome and decreased CYP2D6 enzyme activity^13^. Since *CYP2D6* only partly contributes to explaining the 42.3% variability of endoxifen concentrations^14^ and 68.7% of endoxifen formation (metabolic ratio (MR) of NDM-tamoxifen/endoxifen)^15^, *CYP2D6* genotyping has not been implemented in the daily clinical practice in order to predict tamoxifen efficacy^3,4^. However, other polymorphisms in other drug-metabolizing enzymes involved in tamoxifen metabolism might also have an impact in the endoxifen formation and potentially in clinical outcome^8,16^.

*CYP2C19* gene is highly polymorphic^17^, and it plays multiple roles in the tamoxifen pathway (Fig. 1)^8^. Several polymorphisms in the gene encoding the CYP2C19 enzyme have been described. While CYP2C19*17 variant leads to an increased enzymatic activity^8,16^, other variants e.g. CYP2C19*2 and CYP2C19*3^17^ genotypes have a decreased enzyme activity^8,16^. Regarding the role of these *CYP2C19* genotypes and tamoxifen metabolism, several studies have been published. Lim and colleagues reported no association between tamoxifen and its metabolites concentration levels and *CYP2C19* genotypes^18^. In line with these outcomes, Mürdter et al*.* failed to find an association regarding *CYP2C19* genotypes and endoxifen, 4-hydroxy-tamoxifen and NDM-tamoxifen concentrations or MRs. In contrast, Gjerde et al*.* observed a higher 4-hydroxy-tamoxifen formation in CYP2C19*17 carriers^19^. Interestingly, Lim and colleagues reported in a recent study an association of CYP2C19*2 with norendoxifen, also named 4-hydroxy-N,N-didesmethyltamoxifen^20^. Norendoxifen is an active metabolite of tamoxifen, which is the result of the direct de-methylation of endoxifen. In contrast to endoxifen and tamoxifen, Lu et al*.* characterized this metabolite as dual aromatase inhibitor and selective estrogen-receptor modulator^21^ which may lead to an interesting novel drug^22^.

Also, the relationship between *CYP2C19* genotypes and breast cancer recurrence has been examined, yet contradictory results have also been published. Schroth and colleagues described a more favorable survival outcome for CYP2C19*17 carriers (Hazard Ratio (HR):0.45; 95% Confidence Interval (CI) 0.21–0.92; *p* value 0.03)^23^. Similarly, a meta-analysis described improved survival outcomes in CYP2C19*17 carriers^24^. However, Moyer failed to find an association between clinical outcome and CYP2C19*17 genotype (HR: 0.93; 95% CI 0.64–1.37; *p* value 0.667)^25^. In line with Moyer, these results were recently ratified by Damkier and colleagues after analyzing the publicly available dataset of the International Tamoxifen Pharmacogenomics Consortium (ITPC)^26^. In this heterogeneous group, homo- and heterozygotes of the CYP2C19*17 variant were not associated with better survival outcome.

In the same manner, CYP2C19*2 genotype has been studied, and conflicting results were found again. Schaik and colleagues reported better clinical outcomes in the advanced setting (HR: 0.72; 95% CI 0.57–0.90; *p* value: 0.004) in a cohort of 499 patients^27^. In the same line, Beelen observed better survival results in adjuvant tamoxifen-treated group (HR: 0.26; 95% CI: 0.12–0.55; *p* value: 0.001)^28^, which is accordance with Ruiter and colleagues^29^. In contrast, Damkier showed again no association between CYP2C19*2 genotype and breast cancer outcomes in a larger group of patientsle 2.

Interestingly, another approach to evaluate the effect of *CYP2C19* genotypes was also performed by Schroth and colleagues and later reproduced by Damkier and colleagues^26^. In their studies, patients were categorized according to their *CYP2D6* and *CYP2C19* genotypes in three tamoxifen activity groups (high, intermediate and low). While Schroth reported differences in clinical outcomes across these groups, Damkier failed to find any type of association. An important limitation in the majority of these studies might have been the analysis of each allele in isolation from the other one. Due to the particularities of the *CYP2C19* gene, a better approach might be the use of the real *CYP2C19* genotypes. For instance, a CYP2C19*2/*17 individual illustrates this relevant limitation. In case this patient were studied for CYP2C19*2 genotype only, the actual *CYP2C19* effect would be masked by other genotypes, e.g. CYP2C19*17^30,31^.

Due to this large variety in results, we aimed to investigate the role of *CYP2C19* genotypes on tamoxifen metabolism breast cancer survival outcomes in the large cohort of the prospective CYPTAM study^32^, which enrolled 667 Caucasian pre- and post-menopausal patients diagnosed with early-breast cancer receiving adjuvant tamoxifen.

## Methods

### Study objectives

The primary objective of this study was to investigate the impact of CYP2C19*2 and CYP2C19*17 on the concentrations and MRs of tamoxifen, NDM-tamoxifen, 4-hydroxy-tamoxifen and endoxifen. To this end, patients were classified according to their *CYP2C19* genotypes in six possible groups: CYP2C19*1/*1; CYP2C19*1/*2; CYP2C19*2/*2; CYP2C19*1/*17; CYP2C19*17/*17; CYP2C19*2/*17. At the same time, we also performed another analysis in which we evaluated the effect of CYP2C19*2 (CYP2C19*1/*1 and CYP2C19*1/*2 versus CYP2C19*2/*2) and CYP2C19*17 (CYP2C19*1/*1 and CYP2C19*1/*17 versus CYP2C19*17/*17) separately. However, tamoxifen metabolism is complex and mainly determined by *CYP2D6*, and accounting only for CYP2C19*2 and CYP2C19*17 would not be of significant value, since these genotypes have a minor effect on tamoxifen variability^8,16,33^. Accordingly, tamoxifen activity groups based on the actual *CYP2C19* and *CYP2D6* genotypes were made, and individuals could be categorized in the low, intermediate or high activity group (Table 1).

**Table 1:** Overall Tamoxifen enzymatic activity groups according to *CYP2D6* and *CYP2C19* genotypes.

	CYP2D6*	*CYP2C19*
**Activity groups according to** ***CYP2D6*** **and** ***CYP2C19*** **genotypes**		
High activity	EM/EM	*17/*17; *1/*17
Intermediate activity	EM/EM	*1/*1; *1/*2; *2/*2; *2/*17
	EM/IM	*1/*1; *1/*17; *17/*17; *2/*17
	EM/PM	*1/*1; *1/*17; *17/*17; *2/*17
Low activity	EM/IM	*1/*2; *2/*2
	EM/PM	*1/*2; *2/*2
	IM/IM	*1/*1; *1/*2; *2/*2; *1/*17; *17/*17; *2/*17
	IM/PM	*1/*1; *1/*2; *2/*2; *1/*17; *17/*17; *2/*17
	PM/PM	*1/*1; *1/*2; *2/*2; *1/*17; *17/*17; *2/*17

*EM* extensive metabolizers, *IM* intermediate metabolizer, *PM* poor metabolizer.

*CYP2D6 ultra-metabolizers (UM) were classified as individuals of the high activity group.

The secondary objective was to assess the effect of these two *CYP2C19* variants with breast cancer outcomes in a large cohort of Caucasian patients diagnosed with early-breast cancer receiving adjuvant tamoxifen. In the core CYPTAM study, the selected primary endpoint was relapse-free survival (RFS), which was defined as the time from enrolment to loco-regional or distant relapse or second breast cancer. In case of a switch to an aromatase inhibitor, patients were censored at the moment of tamoxifen discontinuation^32^.

### Study design and population

To research the influence of *CYP2C19* variants on tamoxifen metabolism and survival outcomes, whole blood and serum samples and clinical information and follow-up regarding pre- and post-menopausal female patients encompassed in the CYPTAM study were used. Concisely, from February 2008 till December 2010, a total of 667 patients were enrolled in this study, which comprises research from 25 hospitals from Belgium and The Netherlands. The primary objective was to associate *CYP2D6* predicted phenotypes and endoxifen serum concentration to breast cancer recurrence^32^. In this study, female individuals diagnosed with early-breast cancer and starting 20 mg QD tamoxifen as adjuvant endocrine therapy, were eligible to participate in this observational study. Also, patients were allowed to participate if they were receiving tamoxifen for no longer than twelve months. Exclusion criteria were pregnancy, breast-feeding and earlier malignancy, with the exception of adequately treated in-situ cervix carcinoma and basal-cell carcinoma. The study protocol was approved by the Institutional Review Board of the Leiden University Medical Center (The Netherlands) in accordance with the Declaration of Helsinki (Tokyo 2004) and registered in the Netherlands Trial Registry (NTR1509). All encompassed female individuals gave written informed consent. For this pharmacogenetic study, the CYPTAM population was analysed, which is described in more detail elsewhere^14,34^.

### Quantification analysis and genotyping

Serum and whole blood specimens were collected for quantification analysis of tamoxifen and its metabolites and genotyping, respectively. Samples were retrieved after at least two-month of tamoxifen therapy in order to assure steady-state concentrations. Also, a minimum of twelve hours after the last intake was required for steady state trough concentrations.

Quantification of tamoxifen and its metabolites concentrations were performed by high-performance liquid chromatography-tandem mass spectrometry (HPLC–MS/MS)^35^. *CYP2D6* Genotyping was performed with Amplichip CYP450 test (Roche Diagnostic, Indianapolis, USA). In accordance with their *CYP2D6* genotypes, all individuals were classified in predicted phenotypes, as defined by Schroth et al. More comprehensive description about *CYP2D6* predicted phenotypes is outlined elsewhere. In the same manner, *CYP2C19* genotyping was performed with Amplichip CYP450 test (Roche Diagnostic, Indianapolis, USA) for CYP2C19*2 and TaqMan7500 (Applied Biosystems, Nieuwerkerk a.d. IJssel, The Netherlands) SNP Genotyping Assays for CYP2C19*17.

Due to the low allele frequency of other *CYP2C19* genotypes in the Caucasian population, no other genotypes were assessed in this study. For instance, CYP2C19*3 variant has a reported frequency of occurrence of 0.04%, while it has an allele frequency of 5–11% in Asian population groups^36,37^. Therefore, only the two most common of *CYP2C19* variants among Caucasians, CYP2C19*2 and CYP2C19*17, were investigated.

### Statistical analysis

To evaluate the role of *CYP2C19* genotypes on tamoxifen metabolism, concentrations and metabolic ratios (MRs) of tamoxifen, endoxifen, NDM-tamoxifen and 4-hydroxy-tamoxifen were used. In this case, MRs were considered as concentration of substrate divided by metabolite concentration. To assess differences between groups, analysis of variance (ANOVA) test were carried out. Also, multiple linear regression analyses were performed to investigate the contributions of these *CYP2C19* genotypes to the total explained variability of MRs and concentrations of tamoxifen, endoxifen, NDM-tamoxifen and 4-hydroxy-tamoxifen.

For the second objective, Cox regression was carried to analyse whether RFS varied across all groups [Hazard Ratio: HR; 95% Confidence Interval (CI)]. When in the univariable analysis, a *p* value below 0.1 was obtained, this covariate was adopted in the multivariable analysis. In addition, the following covariates were fitted in the multivariate analysis due to their known clinical relevance: tumor and nodal stage, histological classification and grade, and Her2 receptor status and menopausal status. Since the variable menopausal status was not available, a surrogate variable based on age at enrolment was used. Premenopausal and postmenopausal patients had an age at enrolment of ≤ 45 years and ≥ 45 years, respectively.

At the same time, we conducted an exploratory examination by performing classification tree analyses in order to determine the levels of interactions by polymorphisms (*CYP2D6* and *CYP2C19* genotypes) on the effect of endoxifen concentrations. More detailed information about how these type of analyses are performed is described elsewhere^38^. All statistical analyses were performed with IBM SPSS for Windows, Version 23.0. Statistical significance was accepted for *p* values below 0.05.

## Results

### Allele frequencies and distributions: *CYP2C19* genotypes

The genotype distributions of *CYP2C19* variants are described in Table 2. In this study, both genotypes were found to be in consistency with Hardy–Weinberg equilibrium (CYP2C19*2: χ^2^ = 0.518, *p* value = 0.472; CYP2C19*17: χ^2^ = 0.135, *p* value = 0.713). Also, a summary of the overall tamoxifen activity groups depending on *CYP2C19* variants and *CYP2D6* genotypes is shown in Table 2. Of note, *CYP2D6* ultra-rapid metabolizers (n = 5) were included in the high activity group, independently of the *CYP2C19* genotype. As illustration, we also divided the CYPTAM patients according to the previously proposed overall tamoxifen activity groups based on *CYP2C19* and *CYP2D6* genotypes by Schroth and colleagues and later reproduced by Damkier and colleagues^26^. An overview of these groups (low, intermediate and high) is listed as Supplementary Table 2.

**Table 2:** Genotype distribution and frequency in the study population.

	Total patients (N)	Frequency (%)
***CYP2C19 genotypes***		
*1/*1	375	57.6
*1/*2	14	2.2
*2/*2	19	2.9
*1/*17	165	25.3
*17/*17	31	4.8
*2/*17	47	7.2
CYP2C19*2		
*1/*1 and *1/*2	635	97.1
*2/*2	19	2.9
CYP2C19*17		
*1/*1 and *1/*17	602	95.1
*17/*17	31	4.9
**Activity groups according to** ***CYP2D6*** **and** ***CYP2C19*** genotypes*		
High activity	67	10.6
Intermediate activity	446	70.6
Low activity	119	18.8

*In total, five *CYP2D6* UM were identified and classified in the high activity groups, independently of *CYP2C19* genotype.

### Study population

Baseline characteristics of the CYPTAM study by *CYP2C19* genotypes (CYP2C19*1/*1; CYP2C19*1/*2; CYP2C19*2/*2; CYP2C19*1/*17; CYP2C19*17/*17; CYP2C19*2/*17) is presented in Table 3. At baseline, no statistically significant differences were found regarding tumor stage, histological classification and grade, progesterone status, type of surgery and axillar surgery, adjuvant chemotherapy and radiotherapy and trastuzumab treatment. Similarly, a second overview of the baseline demographics by CYP2C19*2 and CYP2C19*17 separately, is shown as Supplementary Table 3. At enrolment, all groups were found to be comparable since no statistical differences were observed (Supplementary Table 3). Also, an overview of the demographics at enrolment by the proposed overall tamoxifen activity groups based on *CYP2D6* and *CYP2C19* genotypes is listed as Supplementary Table 4. In the same manner, all groups were similar at baseline, with the exception of nodal stage (*p* value: 0.038).

**Table 3:** Baseline clinical characteristics of the CYPTAM patients according to *CYP2C19* genotypes.

	*CYP2C19* genotypes						
	*1/*1 (N = 375)	*1/*2 (N = 14)	*2/*2 (N = 19)	*1/*17 (N = 164)	*17/*17 (N = 31)	*2/*17 (N = 47)	*P* value
**Age at enrolment (years)**							
Mean (SD)	56.0 (11.0)	60.3 (9.5)	55.6 (10.2)	55.8 (11.6)	57.0 (9.8)	59.1 (11.7)	0.347
**Tumor stage**							
*T1*							0.840
N	203	10	11	86	13	23	
%	58.7%	2.9%	3.2%	24.9%	3.8%	6.6%	
*T2*							
N	153	4	8	68	16	20	
%	56.9%	1.5%	3.0%	25.3%	5.9%	7.4%	
*T3/T4*							
N	14	0	0	9	2	2	
%	51.9%	0.0%	0.0%	33.3%	7.4%	7.4%	
*Not specified*							
N	5	0	0	2	0	2	
%	55.6%	0.0%	0.0%	22.2%	0.0%	22.2%	
**Nodal stage**							
*N0*							0.037
N	166	11	12	78	16	26	
%	53.7%	3.6%	3.9%	25.2%	5.2%	8.4%	
*N1*							
N	166	3	6	64	9	12	
%	63.8%	1.2%	2.3%	24.6%	3.5%	4.6%	
*N2*							
N	34	0	0	16	2	5	
%	59.6%	0.0%	0.0%	28.1%	3.5%	8.8%	
*N3*							
N	7	0	1	7	4	3	
%	31.8%	0.0%	4.5%	31.8%	18.2%	13.6%	
*Not specified*							
N	2	0	0	0	0	1	
%	66.7%	0.0%	0.0%	0.0%	0.0%	33.3%	
**Histological classification**							
*Ductal adenocarcinoma*							0.662
N	280	11	13	131	22	38	
%	56.6%	2.2%	2.6%	26.5%	4.4%	7.7%	
*Lobular adenocarcinoma*							
N	54	3	3	18	7	6	
%	59.3%	3.3%	3.3%	19.8%	7.7%	6.6%	
*Other*							
N	39	0	3	16	2	2	
%	62.9%	0.0%	4.8%	25.8%	3.2%	3.2%	
*Not specified*							
N	2	0	0	0	0	1	
%	66.7%	0.0%	0.0%	0.0%	0.0%	33.3%	
**Histological grade**							
*G1*							0.357
N	46	6	3	22	5	8	
%	51.1%	6.7%	3.3%	24.4%	5.6%	8.9%	
*G2*							
N	213	7	10	95	19	24	
%	57.9%	1.9%	2.7%	25.8%	5.2%	6.5%	
*G3*							
N	113	1	6	46	6	14	
%	60.8%	0.5%	3.2%	24.7%	3.2%	7.5%	
*Not specified*							
N	3	0	0	2	1	1	
%	42.9%	0.0%	0.0%	28.6%	14.3%	14.3%	
**Progesterone receptor status**							
*Positive*							0.476
N	293	14	14	136	26	35	
%	56.6%	2.7%	2.7%	26.3%	5.0%	6.8%	
*Negative*							
N	77	0	5	26	5	10	
%	62.6%	0.0%	4.1%	21.1%	4.1%	8.1%	
*Not specified*							
N	5	0	0	3	0	2	
%	50.0%	0.0%	0.0%	30.0%	0.0%	20.0%	
**HER2 receptor status**							
*0*							0.003
N	232	6	9	103	24	23	
%	58.4%	1.5%	2.3%	25.9%	6.0%	5.8%	
*1* +							
N	98	4	5	35	6	17	
%	59.4%	2.4%	3.0%	21.2%	3.6%	10.3%	
*2* +							
N	11	4	3	14	0	2	
%	32.4%	11.8%	8.8%	41.2%	0.0%	5.9%	
*3* +							
N	32	0	2	13	1	4	
%	61.5%	0.0%	3.8%	25.0%	1.9%	7.7%	
*Not specified*							
N	2	0	0	0	0	1	
%	66.7%	0.0%	0.0%	0.0%	0.0%	33.3%	
**FISH**							
*Positive (amplification)*							0.745
N	35	0	2	14	1	4	
%	62.5%	0.0%	3.6%	25.0%	1.8%	7.1%	
*Negative*							
N	338	14	17	151	30	42	
%	57.1%	2.4%	2.9%	25.5%	5.1%	7.1%	
*Not specified*							
N	2	0	0	0	0	1	
%	66.7%	0.0%	0.0%	0.0%	0.0%	33.3%	
**Surgery**							
*Mastectomy*							0.503
N	168	2	10	82	16	22	
%	56.0%	0.7%	3.3%	27.3%	5.3%	7.3%	
*Breast conserving*							
N	204	12	9	82	15	24	
%	59.0%	3.5%	2.6%	23.7%	4.3%	6.9%	
*Not specified*							
N	3	0	0	1	0	1	
%	60.0%	0.0%	0.0%	20.0%	0.0%	20.0%	
**Surgery axilla**							
*Sentinal node procedure only*							0.734
N	183	11	9	84	14	24	
%	56.3%	3.4%	2.8%	25.8%	4.3%	7.4%	
*Axillary lymph node dissection*							
N	189	3	10	80	17	22	
%	58.9%	0.9%	3.1%	24.9%	5.3%	6.9%	
*Not specified*							
N	3	0	0	1	0	1	
%	60.0%	0.0%	0.0%	20.0%	0.0%	20.0%	
**Adjuvant radiotherapy**							
*Yes*							0.237
N	260	13	9	111	23	33	
%	57.9%	2.9%	2.0%	24.7%	5.1%	7.3%	
*No*							
N	113	1	10	54	8	13	
%	56.8%	0.5%	5.0%	27.1%	4.0%	6.5%	
*Not specified*							
N	2	0	0	0	0	1	
%	66.7%	0.0%	0.0%	0.0%	0.0%	33.3%	
**Adjuvant chemotherapy**							
*Yes*							0.058
N	235	2	11	103	19	27	
%	59.2%	0.5%	2.8%	25.9%	4.8%	6.8%	
*No*							
N	138	12	8	62	12	19	
%	55.0%	4.8%	3.2%	24.7%	4.8%	7.6%	
*Not specified*							
N	2	0	0	0	0	1	
%	66.7%	0.0%	0.0%	0.0%	0.0%	33.3%	
**Trastuzumab therapy**							
*Yes*							0.466
N	34	0	2	16	1	3	
%	60.7%	0.0%	3.6%	28.6%	1.8%	5.4%	
*No*							
N	338	14	17	148	30	42	
%	57.4%	2.4%	2.9%	25.1%	5.1%	7.1%	
*Not specified*							
N	3	0	0	1	0	2	
%	50.0%	0.0%	0.0%	16.7%	0.0%	33.3%	
**Menopausal status**							
*Premenopausal (age* ≤ *45 years)*							0.370
N	49	2	2	31	3	4	
%	53.8%	2.2%	2.2%	34.1%	3.3%	4.4%	
*Postmenopausal (age* ≥ *45 years)*							
N	326	12	17	133	28	43	
%	58.3%	2.1%	3.0%	23.8%	5.0%	7.7%	

### Associations of tamoxifen and its metabolites concentrations and MRs with *CYP2C19* genotypes

**Figure 2:**
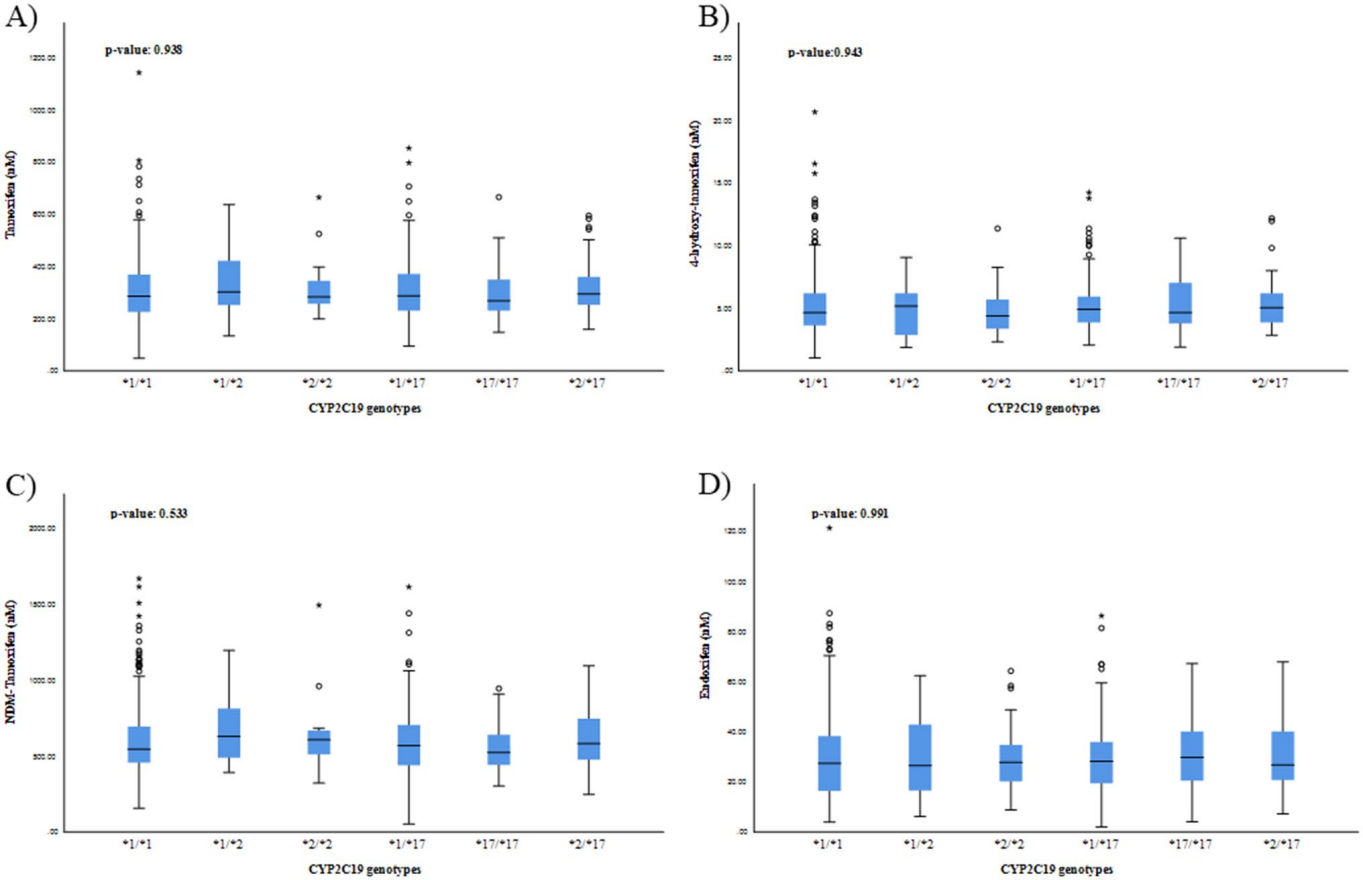
Association of *CYP2C19* genotypes with tamoxifen (**A**), 4-hydroxy-tamoxifen (**B**), and NDM-tamoxifen (**C**) and endoxifen (D) concentration levels. The *CYP2C19* genotypes presented are: CYP2C19*1/*1; CYP2C19*1/*2; CYP2C19*2/*2; CYP2C19*1/*17; CYP2C19*17/*17; CYP2C19*2/*17.

No differences in mean concentrations and MRs of tamoxifen, endoxifen, 4-hydroxy-tamoxifen and NDM-tamoxifen were observed when all *CYP2C19* genotypes were compared (CYP2C19*1/*1; CYP2C19*1/*2; CYP2C19*2/*2; CYP2C19*1/*17; CYP2C19*17/*17; CYP2C19*2/*17). In Fig. 2, mean concentrations of tamoxifen, NDM-tamoxifen, 4-hydroyx-tamoxifen and endoxifen by *CYP2C19* genotypes are presented. In the same way, assessing each polymorphism individually (CYP2C19*1/*1 and CYP2C19*1/*2 versus CYP2C19*2/*2; CYP2C19*1/*1 and CYP2C19*1/*17 versus CYP2C19*17/*17), also reached the same outcomes of no differences in mean concentrations and MRs of tamoxifen and its metabolites. In Supplementary Table 4, mean concentrations and MRs by *CYP2C19* genotypes are listed. In contrast, statistically significant differences in mean concentrations and MRs were found when the overall tamoxifen activity groups (high, intermediate and low) were compared (Supplementary Table 5), with the exception of tamoxifen concentrations. In the same line, analysing the proposed tamoxifen activity groups by Schroth and colleagues and later reproduced by Damkier et al*.*^26^, yielded statistical differences in mean concentrations and MRs (Supplementary Table 6).

To study the additional effect of *CYP2C19* genotypes to the explained variance of tamoxifen and its metabolites concentrations and MRs, these variants were fitted in a multiple regression model in which previously *CYP2D6* genotypes and concentrations of tamoxifen and its metabolites were already assessed^14^. When both *CYP2C19* genotypes were introduced in the model, the explained variability of the concentration levels of tamoxifen and its metabolites barely differed. In Supplementary Table 7, a summary of *CYP2C19* variants covariate analysis is presented.

### Breast cancer recurrence and *CYP2C19* genotypes

**Figure 3:**
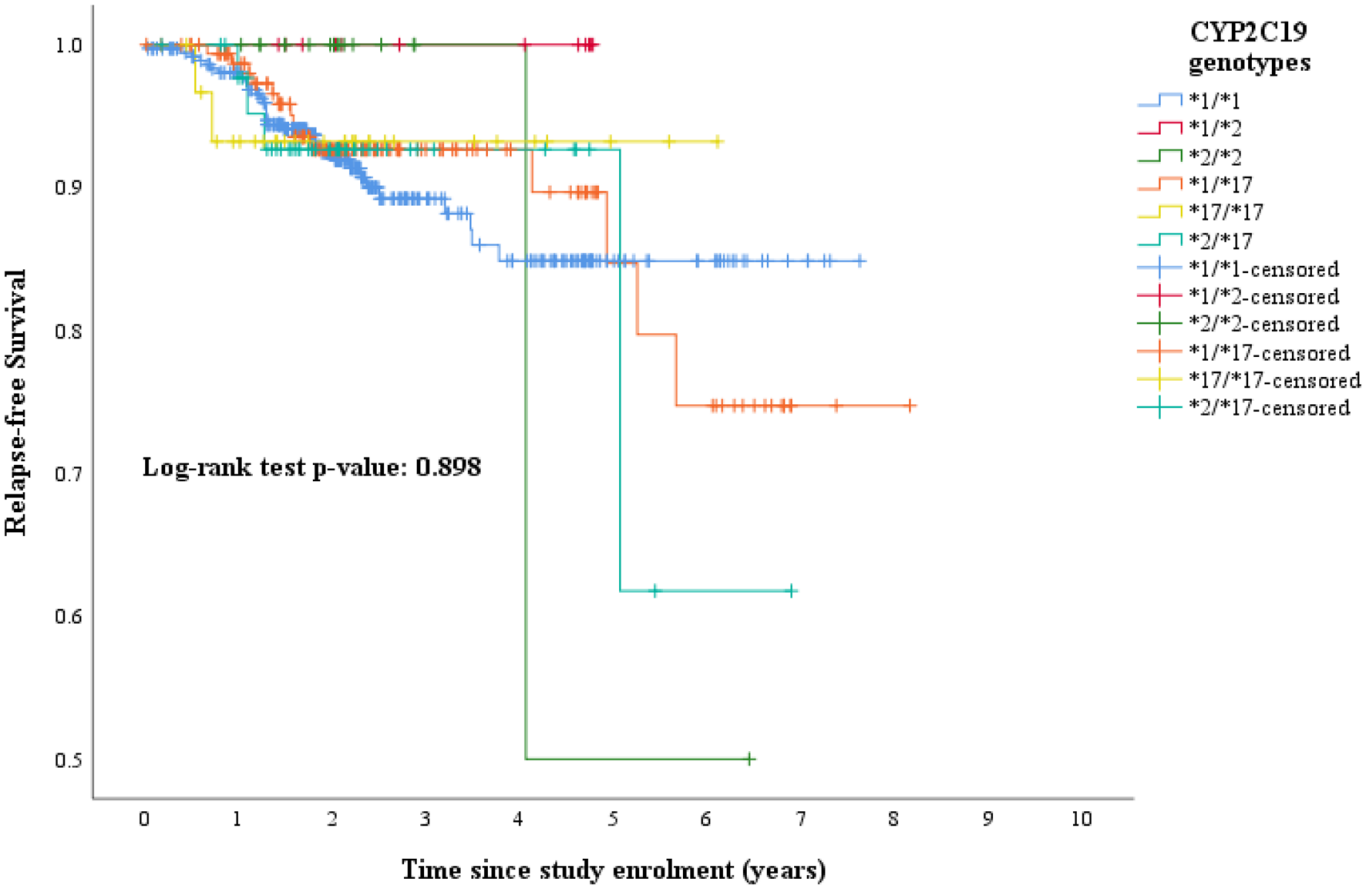
Kaplan–Meier curve for RFS by *CYP2C19* genotypes. Log-rank test *p* value: 0.898. *RFS* Relapse-free survival.

In this study, no differences in terms of HR were observed when comparing all the *CYP2C19* genotypes (CYP2C19*1/*1; CYP2C19*1/*2; CYP2C19*2/*2; CYP2C19*1/*17; CYP2C19*17/*17; CYP2C19*2/*17) (Table 4). In the same line, a log-rank test showed no associations (*p* value: 0.898) across the *CYP2C19* genotypes (Fig. 3). At the same time, evaluating each variants separately did not modify these outcomes (Table 4). Similarly, we did not find any type of association when analysing the proposed tamoxifen activity based on *CYP2D6* and *CYP2C19* genotypes. In Table 4, the uni- and multi-variable Cox regression results are listed. In the same manner, analysing the association between the tamoxifen activity groups, as described by Schroth and Damkier^26^, with RFS, did not modify these outcomes (Supplementary Table 8).

**Table 4:** Cox proportional hazard ratios for *CYP2C19* genotypes, and the proposed tamoxifen activity groups according to *CYP2D6* and *CYP2C19* genotypes.

	Univariable analysis			Multivariable analysis*		
	HR	95% CI	*p* value	HR	95% CI	*p* value
***CYP2C19 genotypes***						
CYP2C19*1/*1	1.000	Reference	(0.923)	1.000	Reference	(0.977)
CYP2C19*1/*2	0.863	0.116–6.434	0.874	0.778	0.098–6.156	0.812
CYP2C19*2/*2	0.657	0.090–4.807	0.679	0.525	0.068–4.039	0.536
CYP2C19*1/*17	0.817	0.490–1.716	0.787	0.883	0.460–1.693	0.707
CYP2C19*17/*17	0.795	0.191–3.314	0.753	0.643	0.146–2.839	0.560
CYP2C19*2/*17	1.066	0.378–3.012	0.904	1.089	0.360–3.097	0.921
CYP2C19*2						
*1/*1 and *1/*2	1.000	Reference		1.000	Reference	
*2/*2	0.755	0.104–5.471	0.781	0.678	0.090–5.131	0.707
CYP2C19*17						
*1/*1 and *1/*17	1.000	Reference		1.000	Reference	
*17/*17	1.896	0.684–5.259	0.219	1.837	0.621–5.441	0.272
**Activity groups according to proposed** ***CYP2D6*** **and** ***CYP2C19*** **genotypes**						
Low activity group	1.000	Reference	(0.174)	1.000	Reference	(0.235)
Intermediate activity group	1.624	0.582–4.531	0.355	1.598	0.561–4.550	0.380
High activity group	0.725	0.194–2.704	0.632	0.759	0.199–2.902	0.687

*Corrected for tumor and nodal stage, histocological classification and grade and Her2Neu receptor status and menopausal status.

### Classification tree analyses

**Figure 4:**
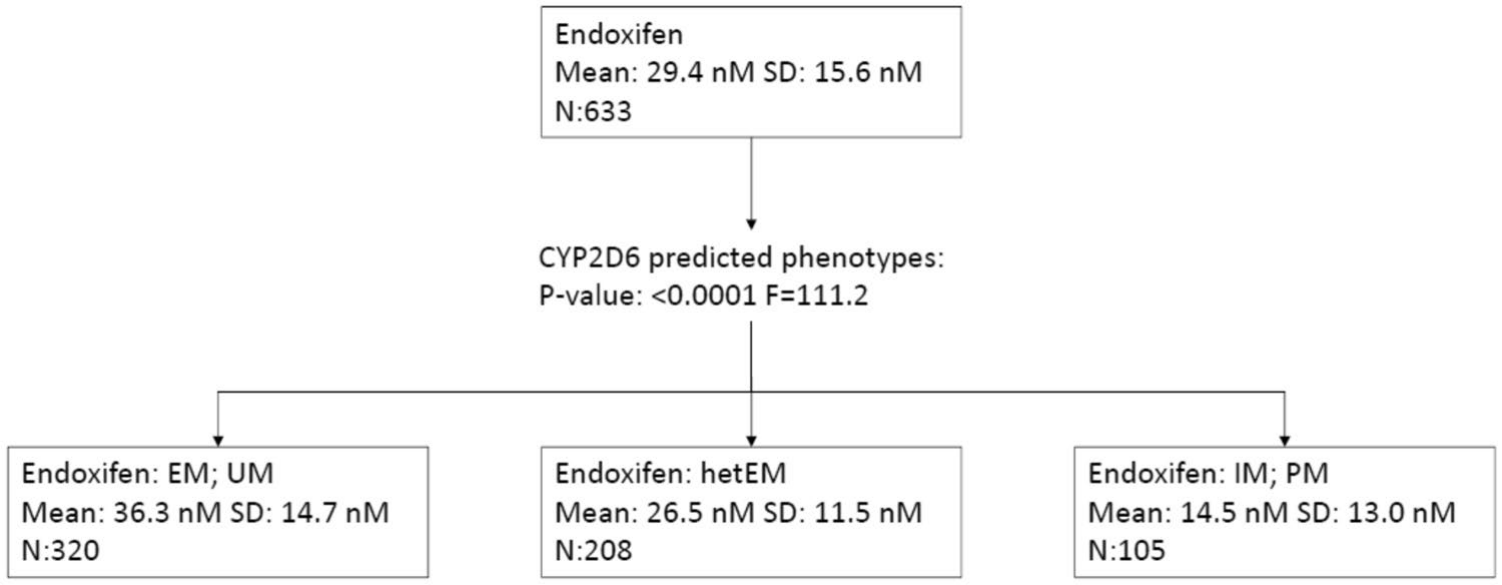
Classification Tree analyses for endoxifen concentrations and *CYP2D6* predicted phenotypes. *EM* extensive metabolizer, *hetEM* heterogenous extensive metabolizer, *IM* intermediate metabolizer, *N* number of individuals, *PM* poor metabolizer, *SD* standard deviation, *UM* ultrarapid metabolizer.

As an exploratory analysis, we conducted different Classification Tree Analyses (CTA) to evaluate the levels of interactions between *CYP2D6* predicted phenotypes and *CYP2C19* genotypes on endoxifen concentrations. The first CTA was performed with the only focus on the *CYP2D6* predicted phenotypes and endoxifen concentrations. In this CTA, patients were subdivided in only one level of the CTA with three different groups of *CYP2D6* phenotypes that statistically different (EM/UM *verus* hetEM *versus* IM/PM; *p* value < 0.001) (Fig. 4). In contrast, adding *CYP2C19* genotypes to this CTA, did not allowed to achieve another level in the CTA.

## Discussion

In this study, we assessed the effect of *CYP2C19* genotypes on tamoxifen metabolism and efficacy in an extensive cohort of Caucasian breast cancer patients receiving tamoxifen as adjuvant endocrine therapy. In our study, we failed to find any differences in mean concentrations and MRs of tamoxifen, endoxifen, 4-hydroxy-tamoxifen and NDMA-tamoxifen when comparing *CYP2C19* genotypes (CYP2C19*1/*1; CYP2C19*1/*2; CYP2C19*2/*2; CYP2C19*1/*17; CYP2C19*17/*17; CYP2C19*2/*17). Additionally, the same outcomes were obtained when each variant was analysed separately. Interestingly, an analysis accounting for *CYP2D6* and *CYP2C19* genotypes, in which the overall tamoxifen enzymatic activity was categorized as high, intermediate and low activity, resulted in statistically significant differences in mean concentrations of endoxifen, NDM-tamoxifen, and 4-hydroxy-tamoxifen and their corresponding MRs. In contrast, tamoxifen mean concentrations were comparable across all the groups. Similarly, we did not find an association between *CYP2C19* genotypes and RFS. In the same manner, dividing patients in low, intermediate or high activity based on their *CYP2D6* and *CYP2C19* genotypes did not show a survival difference.

Tamoxifen has a complex metabolic pathway and many different enzymes are implicated in its activation into endoxifen. Yet, the most relevant enzyme of tamoxifen metabolism is CYP2D6, but it only partially contributes to explaining the inter-variability in endoxifen concentrations between patients. Therefore, many studies have been conducted to find other potentials sources which could clarify the high variability in concentration levels and response to therapy between patients, such as *CYP2C19* genotypes.

According to Schroth and colleagues, the CYP2C19*17/*17 with its higher functioning genotypes has been correlated with improved clinical outcome. In theory, tamoxifen may be more easily metabolized into its active metabolites, e.g. endoxifen, mainly due to the higher enzymatic activity among CYP2C19*17 carriers^19^. Consequently, a higher exposure to the anti-estrogenic activity of tamoxifen and its metabolites could be expected, which potentially may clarify why CYP2C19*17/*17 patients may have an increased survival outcome. However, we also evaluated differences in mean concentrations of tamoxifen and its metabolites, and no differences by *CYP2C19* genotypes were observed. Also, categorizing patients according to their tamoxifen enzymatic activity did not yield any type of associations in our study, nor using the previously proposed groups by Schroth and Damkier^26^. This hypothesis of higher exposure to anti-estrogenic activity due to higher concentration levels of tamoxifen active metabolites was not observed in our study.

Likewise, for the CYP2C19*2 allele, Van Schaik and colleagues^27^, Beelen et al.^28^ and Ruiter and colleagues^29^, found improved survival outcomes in the metastatic setting and in the adjuvant scenario. In this case, the decreased enzymatic activity of CYP2C19*2 may probably lead to a lower exposure to antiestrogenic activity of tamoxifen and its metabolites, due to the potentially lower concentration levels, and therefore, a worsened clinical outcome. Nevertheless, all of these studies reported improved survival outcomes. A potential explanation for this increased clinical outcome among CYP2C19*2 carriers may be due to the increased transformation from endoxifen into norendoxifen, which has a dual antiendocrine mechanism of action^20^. However, we did not find a statistically significant variations in mean concentration levels or MRs. In this case, our results are again in agreement with Damkier and colleagues^26^, still the main advantage of our study might rely on the use of concentration levels.

Following the approach of Schroth, we also created a new combined variable accounting for *CYP2D6* and *CYP2C19* genotypes. However, our classification slightly varied from the one of Schroth and colleagues, since we used the actual *CYP2C19* genotypes. Although the creation of this activity groups is complicated, the use of the real *CYP2C19* genotypes, instead of the simple allele, has a relevant advantage. For instance, a CYP2C19*2/*17 individual could be wrongly classified in the intermediate activity group for the analysis of CYP2C19*2 variant .In contrast, the same patient, would be categorized in the high activity group if the CYP2C19*17 variant was studied. While this interesting difference on enzymatic activity might be critical for the creation of this type of activity groups, the remaining question would be the interpretation of this difference for the clinical practice. Although differences in the stratification could potentially also affect the obtained results, a second analysis following the classification of Schroth did also not show any type of association: no differences in mean concentrations or MRs or clinical outcomes. In this case, we questioned the utility of this type of groups due to the limited role of *CYP2C19* genotypes on tamoxifen metabolism.

To evaluate the rationale after this variable, we conducted a CTA. Interestingly, we failed to find any improvement in the prediction of endoxifen concentrations when *CYP2C19* genotypes were fitted in the corresponding models, whereas only when *CYP2D6* predicted phenotypes were introduced, significant differences were observed. Our interpretation is that the use of *CYP2C19* genotypes only in order to predict endoxifen concentrations, might lack of usefulness in the clinical setting, and that *CYP2D6* genotypes might have the most relevant role in the prediction of endoxifen concentrations. Due to differences in mean concentrations and metabolic ratios when using this type of groups (based on *CYP2D6* and *CYP2C19* genotypes), we hypothesize that *CYP2C19* genotypes might help to compensate the formation of endoxifen and 4-hydroxy-tamoxifen in the case of low CYP2D6 enzymatic activity. Yet, this minor effect did not translate in better clinical outcomes.

A limitation of our study might be our sample size of 667 patients compared to the cohort of 2102 female patients of the ITPC. However, we believe that our study was sufficiently powered to replicate the results of Damkier and colleagues^26^, with the main advantage of the use of concentrations and MRs.

Another possible limitation in our study might the potential effect of *CYP2C19* phenoconversion in cancer patients. While an acquired loss of activity in *CYP2C19* has been described in the literature in cancer patients^39^, the real impact of this discrepancy between *CYP2C19* pheno- and genotype in breast cancer patients receiving adjuvant tamoxifen is unclear. However, we believe that the effect of *CYP2C19* phenoconversion in tamoxifen metabolism might be small. In our opinion, since *CYP2C19* genotypes barely contributes to explaining the inter-patient variability of tamoxifen and its metabolites concentrations and MRs, small differences in concentrations or MRs due to a *CYP2C19* phenoconversion might be unnoticed.

To conclude, we have shown that *CYP2C19* polymorphisms have no or little impact on concentration levels and MRs of tamoxifen, endoxifen, 4-hydroxy-tamoxifen and NDM-tamoxifen, or clinical outcomes in breast cancer patients. Therefore, *CYP2C19* genotypes might not be clinically decisive for patients with early-breast cancer treated with adjuvant tamoxifen.

## Supplementary Information

Supplementary Information.
