# Supplementary material for: Effect of CYP2C19 genotypes on tamoxifen metabolism and early-breast cancer relapse
Source: Sci Rep. 2021 Jan 11;11:415. doi: 10.1038/s41598-020-79972-x (PMC7801676; doi:10.1038/s41598-020-79972-x)
Supplement: Supplementary file 1 — Supplementary Information. [file 41598_2020_79972_MOESM1_ESM.docx]

**Effect of *CYP2C19* genotypes on tamoxifen metabolism and early-breast cancer relapse**

Sanchez-Spitman A.B.^1,2^, Swen J.J.^1,2^, Dezentjé V.O.^3^, Moes D.J.A.R.^1,2^, Gelderblom H.^2,4^, Guchelaar H.J. ^1,2*^

**Affiliations**

^1^ Department of Clinical Pharmacy & Toxicology, Leiden University Medical Center, Leiden, The Netherlands.

^2^ Leiden Network for Personalised Therapeutics, Leiden University Medical Center, Leiden, The Netherlands.

^3^ Department of Medical Oncology, Netherlands Cancer Institute / Antoni van Leeuwenhoek, Amsterdam, The Netherlands.

^4^ Department of Medical Oncology, Leiden University Medical Center, Leiden, The Netherlands

***Corresponding author**

Henk-Jan Guchelaar, PharmD, PhD

Department of Clinical Pharmacy & Toxicology

Leiden University Medical Center

Albinusdreef 2

2300 RC Leiden

The Netherlands

**E-mail:** [**h.j.guchelaar@lumc.nl**](mailto:h.j.guchelaar@lumc.nl)

**Supplementary Table 1.** Overview of the abbreviations used in the manuscript and their meaning

| **Abbreviation** | **Meaning** |
| --- | --- |
| CI | Confidence Interval |
| CTA | Classification Tree Analysis |
| EM | Extensive metabolizer |
| HR | Hazard Ratio |
| HetEM | Heterozygous extensive metabolizer |
| IM | Intermediate metabolizer |
| MR | Metabolic Ratio |
| NDM-tamoxifen | N-desmethyl-tamoxifen |
| PM | Poor metabolizer |
| RFS | Relapse-free survival during tamoxifen treatment |
| SD | Standard Deviation |
| UM | Ultrarapid metabolizer |

**Supplementary Table** 2. Tamoxifen enzymatic activity groups as proposed by Schroth^1,2^ and Damkier^3^ and based on *CYP2D6* predicted phenotypes and *CYP2C19*2* and *CYP2C19*17* genotypes and their distribution and frequency in the CYPTAM cohort.

|  | | ***CYP2D6*** | ****17*** | **Total patients (N)** | **Frequency (%)** |
| --- | --- | --- | --- | --- | --- |
| **Activity groups according to CYP2D6 predicted phenotypes and *CYP2C19*2* genotype** | **High activity** | **EM/EM** | **Yes** | 155 | 24.4 |
|  | **Intermediate activity** | **EM/EM** | **No** | 281 | 44.3 |
|  |  | **EM/IM** | **Yes** |  |  |
|  |  | **EM/PM** | **Yes** |  |  |
|  | **Low activity** | **EM/IM** | **No** | 198 | 31.2 |
|  |  | **EM/PM** | **No** |  |  |
|  |  | **IM/IM** | **Yes or No** |  |  |
|  |  | **IM/PM** | **Yes or No** |  |  |
|  |  | **PM/PM** | **Yes or No** |  |  |
|  |  | ***CYP2D6*** | ****2*** |  |  |
| **Activity groups according to CYP2D6 predicted phenotypes and *CYP2C19*17* genotype** | **High activity** | **EM/EM** | **No** | 79 | 12.8 |
|  | **Intermediate activity** | **EM/EM** | **Yes** | 249 | 40.3 |
|  |  | **EM/IM** | **No** |  |  |
|  |  | **EM/PM** | **No** |  |  |
|  | **Low activity** | **EM/IM** | **Yes** | 290 | 46.9 |
|  |  | **EM/PM** | **Yes** |  |  |
|  |  | **IM/IM** | **Yes or No** |  |  |
|  |  | **IM/PM** | **Yes or No** |  |  |
|  |  | **PM/PM** | **Yes or No** |  |  |

*Ultra-metabolizers (UM) were treated as extensive metabolizers (EM). EM: extensive metabolizers; IM: intermediate metabolizer; PM: poor metabolizer.

**Supplementary Table 3.** Baseline clinical characteristics of the CYPTAM patients according *CYP2C19*2* and *CYP2C19*17* genotypes

|  | | | ***CYP2C19*2*** | | | ***CYP2C19*17*** | | |
| --- | --- | --- | --- | --- | --- | --- | --- | --- |
|  |  |  | ****1/*1* and **1/*2*** | ****2/*2*** | **p-value** | ****1/*1* and **1/*17*** | ****17/*17*** | **p-value** |
| **Age at enrolment (years)** | | **Mean (SD)** | 56.4 (11.1) | 54.5 (9.7) | 0.650 | 56.3 (11.1) | 57.3 (10.3) | 0.628 |
| **Tumor stage** | **T1** | **N** | 333 | 12 | 0.569 | 323 | 16 | 0.761 |
|  |  | **%** | 96.5% | 3.5% |  | 95.3% | 4.7% |  |
|  | **T2** | **N** | 265 | 6 |  | 246 | 12 |  |
|  |  | **%** | 97.8% | 2.2% |  | 95.3% | 4.7% |  |
|  | **T3/T4** | **N** | 28 | 0 |  | 25 | 2 |  |
|  |  | **%** | 100.0% | 0.0% |  | 92.6% | 7.4% |  |
|  | **Not specified** | **N** | 9 | 0 |  | 8 | 1 |  |
|  |  | **%** | 100.0% | 0.0% |  | 88.9% | 11.1% |  |
| **Nodal stage** | **N0** | **N** | 298 | 13 | 0.230 | 284 | 18 | 0.188 |
|  |  | **%** | 95.8% | 4.2% |  | 94.0% | 6.0% |  |
|  | **N1** | **N** | 256 | 4 |  | 243 | 9 |  |
|  |  | **%** | 98.5% | 1.5% |  | 96.4% | 3.6% |  |
|  | **N2** | **N** | 55 | 0 |  | 52 | 1 |  |
|  |  | **%** | 100.0% | 0.0% |  | 98.1% | 1.9% |  |
|  | **N3** | **N** | 23 | 1 |  | 20 | 3 |  |
|  |  | **%** | 95.8% | 4.2% |  | 87.0% | 13.0% |  |
|  | **Not specified** | **N** | 3 | 0 |  | 3 | 0 |  |
|  |  | **%** | 100.0% | 0.0% |  | 100.0% | 0.0% |  |
| **Histological classification** | **Ductal adenocarcinoma** | **N** | 483 | 13 | 0.968 | 464 | 22 | 0.764 |
|  |  | **%** | 97.4% | 2.6% |  | 95.5% | 4.5% |  |
|  | **Lobular adenocarcinoma** | **N** | 90 | 3 |  | 79 | 6 |  |
|  |  | **%** | 96.8% | 3.2% |  | 92.9% | 7.1% |  |
|  | **Other** | **N** | 59 | 2 |  | 56 | 3 |  |
|  |  | **%** | 96.7% | 3.3% |  | 94.9% | 5.1% |  |
|  | **Not specified** | **N** | 3 | 0 |  | 3 | 0 |  |
|  |  | **%** | 100.0% | 0.0% |  | 100.0% | 0.0% |  |
| **Histological grade** | **G1** | **N** | 86 | 3 | 0.886 | 86 | 4 | 0.647 |
|  |  | **%** | 96.6% | 3.4% |  | 95.6% | 4.4% |  |
|  | **G2** | **N** | 363 | 9 |  | 340 | 16 |  |
|  |  | **%** | 97.6% | 2.4% |  | 95.5% | 4.5% |  |
|  | **G3** | **N** | 179 | 6 |  | 170 | 10 |  |
|  |  | **%** | 96.8% | 3.2% |  | 94.4% | 5.6% |  |
|  | **Not specified** | **N** | 7 | 0 |  | 6 | 1 |  |
|  |  | **%** | 100.0% | 0.0% |  | 85.7% | 14.3% |  |
| **Progesterone receptor status** | **Positive** | **N** | 505 | 15 | 0.844 | 481 | 24 | 0.667 |
|  |  | **%** | 97.1% | 2.9% |  | 95.2% | 4.8% |  |
|  | **Negative** | **N** | 121 | 3 |  | 111 | 7 |  |
|  |  | **%** | 97.6% | 2.4% |  | 94.1% | 5.9% |  |
|  | **Not specified** | **N** | 9 | 0 |  | 10 | 0 |  |
|  |  | **%** | 100.0% | 0.0% |  | 100.0% | 0.0% |  |
| **HER2 receptor status** | **0** | **N** | 389 | 10 | 0.805 | 361 | 22 | 0.549 |
|  |  | **%** | 97.5% | 2.5% |  | 94.3% | 5.7% |  |
|  | **1+** | **N** | 159 | 5 |  | 155 | 6 |  |
|  |  | **%** | 97.0% | 3.0% |  | 96.3% | 3.7% |  |
|  | **2+** | **N** | 32 | 2 |  | 34 | 0 |  |
|  |  | **%** | 94.1% | 5.9% |  | 100.0% | 0.0% |  |
|  | **3+** | **N** | 52 | 1 |  | 49 | 3 |  |
|  |  | **%** | 98.1% | 1.9% |  | 94.2% | 5.8% |  |
|  | **Not specified** | **N** | 3 | 0 |  | 3 | 0 |  |
|  |  | **%** | 100.0% | 0.0% |  | 100.0% | 0.0% |  |
| **FISH** | **Positive (amplification)** | **N** | 56 | 1 | 0.850 | 53 | 3 | 0.914 |
|  |  | **%** | 98.2% | 1.8% |  | 94.6% | 5.4% |  |
|  | **Negative** | **N** | 576 | 17 |  | 546 | 28 |  |
|  |  | **%** | 97.1% | 2.9% |  | 95.1% | 4.9% |  |
|  | **Not specified** | **N** | 3 | 0 |  | 3 | 0 |  |
|  |  | **%** | 100.0% | 0.0% |  | 100.0% | 0.0% |  |
| **Surgery** | **Mastectomy** | **N** | 293 | 10 | 0.697 | 275 | 14 | 0.874 |
|  |  | **%** | 96.7% | 3.3% |  | 95.2% | 4.8% |  |
|  | **Breast conserving** | **N** | 337 | 8 |  | 322 | 17 |  |
|  |  | **%** | 97.7% | 2.3% |  | 95.0% | 5.0% |  |
|  | **Not specified** | **N** | 5 | 0 |  | 5 | 0 |  |
|  |  | **%** | 100.0% | 0.0% |  | 100.0% | 0.0% |  |
| **Surgery axilla** | **Sentinal node procedure only** | **N** | 317 | 9 | 0.931 | 298 | 20 | 0.248 |
|  |  | **%** | 97.2% | 2.8% |  | 93.7% | 6.3% |  |
|  | **Axillary lymph node dissection** | **N** | 313 | 9 |  | 299 | 11 |  |
|  |  | **%** | 97.2% | 2.8% |  | 96.5% | 3.5% |  |
|  | **Not specified** | **N** | 5 | 0 |  | 5 | 0 |  |
|  |  | **%** | 100.0% | 0.0% |  | 100.0% | 0.0% |  |
| **Adjuvant radiotherapy** | **Yes** | **N** | 441 | 8 | 0.068 | 417 | 21 | 0.903 |
|  |  | **%** | 98.2% | 1.8% |  | 95.2% | 4.8% |  |
|  | **No** | **N** | 191 | 10 |  | 182 | 10 |  |
|  |  | **%** | 95.0% | 5.0% |  | 94.8% | 5.2% |  |
|  | **Not specified** | **N** | 3 | 0 |  | 3 | 0 |  |
|  |  | **%** | 100.0% | 0.0% |  | 100.0% | 0.0% |  |
| **Adjuvant chemotherapy** | **Yes** | **N** | 389 | 11 | 0.957 | 363 | 20 | 0.841 |
|  |  | **%** | 97.3% | 2.8% |  | 94.8% | 5.2% |  |
|  | **No** | **N** | 243 | 7 |  | 236 | 11 |  |
|  |  | **%** | 97.2% | 2.8% |  | 95.5% | 4.5% |  |
|  | **Not specified** | **N** | 3 | 0 |  | 3 | 0 |  |
|  |  | **%** | 100.0% | 0.0% |  | 100.0% | 0.0% |  |
| **Trastuzumab therapy** | **Yes** | **N** | 56 | 1 | 0.811 | 53 | 3 | 0.868 |
|  |  | **%** | 98.2% | 1.8% |  | 94.6% | 5.4% |  |
|  | **No** | **N** | 573 | 17 |  | 544 | 28 |  |
|  |  | **%** | 97.1% | 2.9% |  | 95.1% | 4.9% |  |
|  | **Not specified** | **N** | 6 | 0 |  | 5 | 0 |  |
|  |  | **%** | 100.0% | 0.0% |  | 100.0% | 0.0% |  |
| **Menopausal status** | **Premenopausal**  **(age ≤ 45 yearss)** | **N** | 89 | 2 | 0.724 | 85 | 2 | 0.225 |
|  |  | **%** | 97.8% | 2.2% |  | 97.7% | 2.3% |  |
|  | **Postmenopausal (age ≥ 45 years)** | **N** | 545 | 16 |  | 516 | 29 |  |
|  |  | **%** | 97.1% | 2.9% |  | 94.7% | 5.3% |  |

**Supplementary Table 4.** Baseline clinical characteristics of the CYPTAM patients according to the proposed tamoxifen overall activity groups based on *CYP2D6* and *CYP2C19* genotypes

|  | | | **Activity groups** | | |  |
| --- | --- | --- | --- | --- | --- | --- |
|  |  |  | **High activity (N=67)** | **Intermediate activity (N=446)** | **Low activity (N=119)** | **P-value** |
| **Age at enrolment (years)** | **Mean (SD)** | | 56.95 (12.0) | 56.25 (10.9) | 55.75 (11.3) | 0.773 |
| **Tumor stage** | **T1** | **N** | 30 | 237 | 67 | 0.209 |
|  |  | **%** | 9.0% | 71.0% | 20.1% |  |
|  | **T2** | **N** | 29 | 184 | 49 |  |
|  |  | **%** | 11.1% | 70.2% | 18.7% |  |
|  | **T3/T4** | **N** | 6 | 18 | 3 |  |
|  |  | **%** | 22.2% | 66.7% | 11.1% |  |
|  | **Not specified** | **N** | 2 | 7 | 0 |  |
|  |  | **%** | 22.2% | 77.8% | 0.0% |  |
| **Nodal stage** | **N0** | **N** | 33 | 193 | 68 | 0.038 |
|  |  | **%** | 11.2% | 65.6% | 23.1% |  |
|  | **N1** | **N** | 23 | 200 | 34 |  |
|  |  | **%** | 8.9% | 77.8% | 13.2% |  |
|  | **N2** | **N** | 6 | 37 | 13 |  |
|  |  | **%** | 10.7% | 66.1% | 23.2% |  |
|  | **N3** | **N** | 5 | 13 | 4 |  |
|  |  | **%** | 22.7% | 59.1% | 18.2% |  |
|  | **Not specified** | **N** | 0 | 3 | 0 |  |
|  |  | **%** | 0.0% | 100.0% | 0.0% |  |
| **Histological classification** | **Ductal adenocarcinoma** | **N** | 52 | 335 | 94 | 0.781 |
|  |  | **%** | 10.8% | 69.6% | 19.5% |  |
|  | **Lobular adenocarcinoma** | **N** | 11 | 63 | 14 |  |
|  |  | **%** | 12.5% | 71.6% | 15.9% |  |
|  | **Other** | **N** | 4 | 45 | 11 |  |
|  |  | **%** | 6.7% | 75.0% | 18.3% |  |
|  | **Not specified** | **N** | 0 | 3 | 0 |  |
|  |  | **%** | 0.0% | 100.0% | 0.0% |  |
| **Histological grade** | **G1** | **N** | 7 | 61 | 17 | 0.881 |
|  |  | **%** | 8.2% | 71.8% | 20.0% |  |
|  | **G2** | **N** | 41 | 253 | 63 |  |
|  |  | **%** | 11.5% | 70.9% | 17.6% |  |
|  | **G3** | **N** | 19 | 127 | 37 |  |
|  |  | **%** | 10.4% | 69.4% | 20.2% |  |
|  | **Not specified** | **N** | 0 | 5 | 2 |  |
|  |  | **%** | 0.0% | 71.4% | 28.6% |  |
| **Progesterone receptor status** | **Positive** | **N** | 53 | 352 | 96 | 0.651 |
|  |  | **%** | 10.6% | 70.3% | 19.2% |  |
|  | **Negative** | **N** | 13 | 85 | 23 |  |
|  |  | **%** | 10.7% | 70.2% | 19.0% |  |
|  | **Not specified** | **N** | 1 | 9 | 0 |  |
|  |  | **%** | 10.0% | 90.0% | 0.0% |  |
| **HER2 receptor status** | **0** | **N** | 44 | 268 | 70 | 0.401 |
|  |  | **%** | 11.5% | 70.2% | 18.3% |  |
|  | **1+** | **N** | 10 | 122 | 30 |  |
|  |  | **%** | 6.2% | 75.3% | 18.5% |  |
|  | **2+** | **N** | 5 | 21 | 8 |  |
|  |  | **%** | 14.7% | 61.8% | 23.5% |  |
|  | **3+** | **N** | 8 | 32 | 11 |  |
|  |  | **%** | 15.7% | 62.7% | 21.6% |  |
|  | **Not specified** | **N** | 0 | 3 | 0 |  |
|  |  | **%** | 0.0% | 100.0% | 0.0% |  |
| **FISH** | **Positive (amplification)** | **N** | 9 | 35 | 11 | 0.471 |
|  |  | **%** | 16.4% | 63.6% | 20.0% |  |
|  | **Negative** | **N** | 58 | 408 | 108 |  |
|  |  | **%** | 10.1% | 71.1% | 18.8% |  |
|  | **Not specified** | **N** | 0 | 3 | 0 |  |
|  |  | **%** | 0.0% | 100.0% | 0.0% |  |
| **Surgery** | **Mastectomy** | **N** | 37 | 195 | 59 | 0.267 |
|  |  | **%** | 12.7% | 67.0% | 20.3% |  |
|  | **Breast conserving** | **N** | 30 | 247 | 60 |  |
|  |  | **%** | 8.9% | 73.3% | 17.8% |  |
|  | **Not specified** | **N** | 0 | 4 | 0 |  |
|  |  | **%** | 0.0% | 100.0% | 0.0% |  |
| **Surgery axilla** | **Sentinal node procedure only** | **N** | 31 | 215 | 67 | 0.370 |
|  |  | **%** | 9.9% | 68.7% | 21.4% |  |
|  | **Axillary lymph node dissection** | **N** | 36 | 227 | 52 |  |
|  |  | **%** | 11.4% | 72.1% | 16.5% |  |
|  | **Not specified** | **N** | 0 | 4 | 0 |  |
|  |  | **%** | 0.0% | 100.0% | 0.0% |  |
| **Adjuvant radiotherapy** | **Yes** | **N** | 42 | 312 | 83 | 0.572 |
|  |  | **%** | 9.6% | 71.4% | 19.0% |  |
|  | **No** | **N** | 25 | 131 | 36 |  |
|  |  | **%** | 13.0% | 68.2% | 18.8% |  |
|  | **Not specified** | **N** | 0 | 3 | 0 |  |
|  |  | **%** | 0.0% | 100.0% | 0.0% |  |
| **Adjuvant chemotherapy** | **Yes** | **N** | 42 | 275 | 74 | 0.867 |
|  |  | **%** | 10.7% | 70.3% | 18.9% |  |
|  | **No** | **N** | 25 | 168 | 45 |  |
|  |  | **%** | 10.5% | 70.6% | 18.9% |  |
|  | **Not specified** | **N** | 0 | 3 | 0 |  |
|  |  | **%** | 0.0% | 100.0% | 0.0% |  |
| **Trastuzumab therapy** | **Yes** | **N** | 10 | 34 | 11 | 0.174 |
|  |  | **%** | 18.2% | 61.8% | 20.0% |  |
|  | **No** | **N** | 57 | 406 | 108 |  |
|  |  | **%** | 10.0% | 71.1% | 18.9% |  |
|  | **Not specified** | **N** | 0 | 6 | 0 |  |
|  |  | **%** | 0.0% | 100.0% | 0.0% |  |
| **Menopausal status** | **Premenopausal (age ≤ 45 years)** | **N** | 11 | 57 | 20 | 0.442 |
|  |  | **%** | 12.5% | 64.8% | 22.7% |  |
|  | **Postmenopausal (age ≥ 45 years)** | **N** | 56 | 388 | 99 |  |
|  |  | **%** | 10.3% | 71.5% | 18.2% |  |

**Supplementary Table 5.**Overview of concentration levels and metabolic ratios of tamoxifen, endoxifen. 4-hydroxy-tamoxifen and NDM-tamoxifen by *CYP2C19* genotypes and the proposed tamoxifen activity levels based on *CYP2D6* and *CYP2C19* genotypes. SD: standard deviation; MR: metabolic ratio

|  | |  | **Tamoxifen** | **NDM-Tamoxifen** | **4-Hydroxy-Tamoxifen** | **Endoxifen** | **MR Tamoxifen / NDM-Tamoxifen** | **MR Tamoxifen / 4-Hydroxy-Tamoxifen** | **MR 4-Hydroxy-Tamoxifen / Endoxifen** | **MR NDM-Tamoxifen / Endoxifen** |
| --- | --- | --- | --- | --- | --- | --- | --- | --- | --- | --- |
|  |  | **N** | **Mean (SD)** | **Mean (SD)** | **Mean (SD)** | **Mean (SD)** | **Mean (SD)** | **Mean (SD)** | **Mean (SD)** | **Mean (SD)** |
| ***CYP2C19* genotypes** | ****1/*1*** | 372 | 310.24  (124.91) | 599.90  (224.85) | 5.16  (2.40) | 29.36  (16.82) | 0.53  (0.14) | 65.48  (26.84) | 0.20  (0.09) | 29.91  (27.09) |
|  | ****1/*2*** | 14 | 341.90  (132.84) | 687.34  (255.91) | 5.10  (2.36) | 28.93  (16.43) | 0.50  (0.11) | 74.98  (29.37) | 0.21  (0.11) | 34.50  (27.87) |
|  | ****2/*2*** | 19 | 318.63  (112.40) | 641.61  (242.61) | 4.92  (2.20) | 30.95  (16.31) | 0.50  (0.08) | 71.16  (24.41) | 0.18  (0.07) | 27.40  (18.68) |
|  | ****1/*17*** | 163 | 309.50  (121.89) | 597.93  (233.74) | 5.30  (2.08) | 29.14  (14.50) | 0.53  (0.15) | 62.22  (25.25) | 0.20  (0.08) | 27.26  (21.75) |
|  | ****17/*17*** | 31 | 303.51  (107.50) | 556.73  (166.30) | 5.36  (2.14) | 30.75  (14.19) | 0.56  (0.15) | 62.44  (26.39) | 0.19  (0.07) | 24.73  (20.66) |
|  | ****2/*17*** | 46 | 317.75  (109.48) | 614.33  (194.04) | 5.40  (2.13) | 30.04  (14.54) | 0.53  (0.12) | 63.44  (23.33) | 0.21  (0.09) | 28.10  (22.81) |
|  | **p-value** |  | 0.938 | 0.533 | 0.943 | 0.991 | 0.756 | 0.365 | 0.822 | 0.702 |
| ***CYP2C19*2*** | ****1/*1* and **1/*2*** | 635 | 311.90 (122.79) | 602.73 (225.13) | 5.21 (2.29) | 29.28 (15.93) | 0.53 (0.14) | 65.04  26.71) | 0.20  (0.09) | 29.43  (26.45) |
|  | ****2/*2*** | 19 | 303.84  (105.45) | 606.48  (245.04) | 4.84  (2.07) | 32.35  (15.70) | 0.51  (0.09) | 68.21  23.71) | 0.17  (0.07) | 24.72  (19.16 |
|  | **p-value** |  | 0.783 | 0.945 | 0.507 | 0.421 | 0.664 | 0.618 | 0.083 | 0.454 |
| ***CYP2C19*17*** | ****1/*1* and **1/*17*** | 602 | 309.83  (119.11) | 599.59  (223.20) | 5.16  (2.26) | 29.22  (15.90) | (.53  (0.14) | 65.21  26.61) | 0.20  (0.08) | 29.07  (25.29) |
|  | ****17/*17*** | 31 | 285.16  (80.81) | 558.57  (159.31) | 5.31  (1.90) | 29.77  (14.24) | 0.53  (0.14) | 57.47  17.24) | 0.20  (0.08) | 26.26  (20.64) |
|  | **p-value** |  | 0.255 | 0.313 | 0.713 | 0.849 | 0.997 | 0.110 | 0.907 | 0.544 |
| **Activity groups** | **High activity** | 67 | 283.04  (83.14) | 505.94  (157.44) | 5.83  (2.16) | 34.44  (15.11) | 0.58  (0.14) | 52.85  (21.10) | 0.18  (0.06) | 18.92  (15.60) |
|  | **Intermediate activity** | 441 | 314.78  (126.54) | 593.25  (224.69) | 5.34  (2.37) | 31.15  (15.68) | 0.54  (0.13) | 62.80  (21.28) | 0.19  (0.07) | 24.74  (20.02) |
|  | **Low activity** | 119 | 313.21  (121.36) | 682.12  (223.98) | 4.39  (1.72) | 20.58  (13.35) | 0.47  (0.14) | 78.06  (35.72) | 0.26  (0.11) | 48.62  (33.41) |
|  | **p-value** |  | 0.136 | <0.001 | <0.001 | <0.001 | <0.001 | <0.001 | <0.001 | <0.001 |

**Supplementary Table 6.** Overview of concentration levels and metabolic ratios of tamoxifen, endoxifen, 4-hydroxy-tamoxifen and NDM-tamoxifen based on the *CYP2D6* genotypes and *CYP2C19*2* and *CYP2C19*17* genotypes groups that previously were proposed by Schroth^1,2^ and Damkier^3^. SD: standard deviation; MR: metabolic ratio

|  | |  | **Tamoxifen** | **NDM-Tamoxifen** | **4-Hydroxy-Tamoxifen** | **Endoxifen** | **MR Tamoxifen / NDM-Tamoxifen** | **MR Tamoxifen / 4-Hydroxy-Tamoxifen** | **MR 4-Hydroxy-Tamoxifen / Endoxifen** | **MR NDM-Tamoxifen / Endoxifen** |
| --- | --- | --- | --- | --- | --- | --- | --- | --- | --- | --- |
|  |  | **N** | **Mean (SD)** | **Mean (SD)** | **Mean (SD)** | **Mean (SD)** | **Mean (SD)** | **Mean (SD)** | **Mean (SD)** | **Mean (SD)** |
| **Activity groups according to CYP2D6 predicted phenotypes and *CYP2C19*2* genotype** | **High activity** | 155 | 302.93 (125.40) | 549.08 (219.10) | 5.88 (2.68) | 35.96 (17.38) | 0.56 (0.13) | 55.48 (19.57) | 0.18 (0.07) | 20.83 (21.47) |
|  | **Intermediate activity** | 281 | 311.58 (122.77) | 596.59 (223.28) | 5.17 (2.15) | 29.21 (14.25) | 0.53 (0.12) | 63.77 (21.18) | 0.20 (0.08) | 26.19 (20.25) |
|  | **Low activity** | 198 | 318.40 (119.48) | 653.54 (221.72) | 4.69 (1.96) | 24.34 (14.90) | 0.50 (0.16) | 74.26 (31.98) | 0.23 (0.10) | 40.08 (32.79) |
|  | **p-value** |  | 0.500 | <0.001 | <0.001 | <0.001 | <0.001 | <0.001 | <0.001 | <0.001 |
| **Activity groups according to CYP2D6 predicted phenotypes and *CYP2C19*17* genotype** | **High activity** | 79 | 285.26 (90.48) | 512.08 (182.17) | 5.67 (1.93) | 32.68 (13.31) | 0.58 (0.13) | 53.74 (18.89) | 0.19 (0.08) | 21.25 (21.39) |
|  | **Intermediate activity** | 249 | 312.20 (122.46) | 583.92 (228.08) | 5.50 (2.52) | 33.17 (16.59) | 0.55 (0.13) | 60.87 (22.32) | 0.18 (0.07) | 23.33 (20.69) |
|  | **Low activity** | 290 | 311.88 (119.83) | 631.60 (216.91) | 4.74 (1.98) | 24.89 (14.48) | 0.50 (0.14) | 71.27 (28.88) | 0.22 (0.09) | 36.10 (29.54) |
|  | **p-value** |  | 0.176 | <0.001 | <0.001 | <0.001 | <0.001 | <0.001 | <0.001 | <0.001 |

**Supplementary table 7.** Summary of *CYP2C19* genotypes covariate analysis. Ln(Tamoxifen)= natural log of tamoxifen concentration; Ln(Endoxifen)= natural log of endoxifen concentration; Ln(4-Hydroxy-Tamoxifen)= natural log of 4-hydroxy-tamoxifen concentration; Ln(NDM-Tamoxifen)= natural log of NDM-tamoxifen concentration. MR= Metabolic ratio. Ln(MR Tamoxifen/NDM-Tamoxifen)= natural log of MR Tamoxifen/NDM-Tamoxifen; Ln(MR Tamoxifen/4-hydroxy-tamoxifen)= natural log of MR Tamoxifen/4-hydroxy-tamoxifen; Ln(MR 4-Hydroy-Tamoxifen/Endoxifen)= natural log of MR 4-Hydroy-Tamoxifen/Endoxifen; Ln(MR NDM-Tamoxifen/Endoxifen)= natural log of MR NDM-Tamoxifen/Endoxifen

|  |  | **R^2^** | **p-value** |
| --- | --- | --- | --- |
| **Ln Tamoxifen** | ***CYP2D6*** | 0.003 | 0.169 |
|  | ***CYP2C19*** | 0.003 | 0.157 |
| **Ln Endoxifen** | ***CYP2D6*** | 0.423 | <0.001 |
|  | ***CYP2C19*** | 0.417 | 0.652 |
| **Ln 4-Hydroy-Tamoxifen** | ***CYP2D6*** | 0.127 | <0.001 |
|  | ***CYP2C19*** | 0.123 | 0.272 |
| **Ln NDM-Tamoxifen** | ***CYP2D6*** | 0.138 | <0.001 |
|  | ***CYP2C19*** | 0.143 | 0.730 |
| **Ln MR Tamoxifen/NDM-Tamoxifen** | ***CYP2D6*** | 0.218 | <0.001 |
|  | ***CYP2C19*** | 0.221 | 0.964 |
| **Ln MR Tamoxifen/4-hydroxy-tamoxifen** | ***CYP2D6*** | 0.219 | <0.001 |
|  | ***CYP2C19*** | 0.217 | 0.307 |
| **Ln MR 4-Hydroy-Tamoxifen/Endoxifen** | ***CYP2D6*** | 0.449 | <0.001 |
|  | ***CYP2C19*** | 0.459 | 0.369 |
| **Ln MR NDM-Tamoxifen/Endoxifen** | ***CYP2D6*** | 0.570 | <0.001 |
|  | ***CYP2C19*** | 0.575 | 0.845 |

**Supplementary Table 8.** Cox proportional hazard ratios for tamoxifen overall activity groups previously proposed by Schroth^1,2^ and Damkier^3^ and according to *CYP2D6* predicted phenotypes and *CYP2C19*2* and *CYP2C19*17* genotype. HR: Hazard Ratio; CI: Confidence Interval *Corrected for tumor and nodal stage, histological classification and grade and Her2Neu receptor status and menopausal status.

|  | **Univariable analysis** | | | **Multivariable analysis*** | | |
| --- | --- | --- | --- | --- | --- | --- |
|  | **HR** | **95 % CI** | **p-value** | **HR** | **95 % CI** | **p-value** |
| **Activity groups according to *CYP2D6* predicted phenotypes and *CYP2C19*2* genotype** |  |  |  |  |  |  |
| **Low activity group** | 1.000 | Reference | (0.804) | 1.000 | Reference | (0.704) |
| **Intermediate activity group** | 1.133 | 0.531-2.147 | 0.747 | 1.191 | 0.538-2.636 | 0.666 |
| **High activity group** | 1.246 | 0.648-2.398 | 0.510 | 1.346 | 0.669-2.708 | 0.404 |
|  |  |  |  |  |  |  |
| **Activity groups according to *CYP2D6* predicted phenotypes and *CYP2C19*17* genotype** |  |  |  |  |  |  |
| **Low activity group** | 1.000 | Reference | (0.582) | 1.000 | Reference | (0.458) |
| **Intermediate activity group** | 0.832 | 0.313-2.208 | 0.711 | 0.819 | 0.302-2.220 | 0.695 |
| **High activity group** | 1.271 | 0.707-2.285 | 0.423 | 1.369 | 0.736-2.548 | 0.321 |

**References**

1. Schroth, W.*, et al.* Breast cancer treatment outcome with adjuvant tamoxifen relative to patient CYP2D6 and CYP2C19 genotypes. *J Clin Oncol* **25**, 5187-5193 (2007).

2. Schroth, W.*, et al.* Association between CYP2D6 polymorphisms and outcomes among women with early stage breast cancer treated with tamoxifen. *JAMA* **302**, 1429-1436 (2009).

3. Damkier, P.*, et al.* CYP2C19*2 and CYP2C19*17 variants and effect of tamoxifen on breast cancer recurrence: Analysis of the International Tamoxifen Pharmacogenomics Consortium dataset. *Sci Rep* **7**, 7727 (2017).
